# 2-D Peripheral image quality metrics with different types of multifocal contact lenses

**DOI:** 10.1038/s41598-019-54783-x

**Published:** 2019-12-06

**Authors:** Miguel García García, Siegfried Wahl, Dibyendu Pusti, Pablo Artal, Arne Ohlendorf

**Affiliations:** 1 Eberhard Karls University Tuebingen, Institute for Ophthalmic Research, Tuebingen, 72076 Germany; 2 Carl Zeiss Vision International GmbH, Technology & Innovation, Aalen, 73430 Germany; 3 Universidad de Murcia, Laboratorio de Óptica, Murcia, 30100 Spain

**Keywords:** Visual system, Pattern vision, Translational research, Imaging and sensing

## Abstract

To evaluate the impact of multifocal contact lens wear on the image quality metrics across the visual field in the context of eye growth and myopia control. Two-dimensional cross-correlation coefficients were estimated by comparing a reference image against the computed retinal images for every location. Retinal images were simulated based on the measured optical aberrations of the naked eye and a set of multifocal contact lenses (centre-near and centre-distance designs), and images were spatially filtered to match the resolution limit at each eccentricity. Value maps showing the reduction in the quality of the image through each optical condition were obtained by subtracting the optical image quality from the theoretical physiological limits. Results indicate that multifocal contact lenses degrade the image quality independently from their optical design, though this result depends on the type of analysis conducted. Analysis of the image quality across the visual field should not be oversimplified to a single number but split into regional and groups because it provides more insightful information and can avoid misinterpretation of the results. The decay of the image quality caused by the multifocal contacts alone, cannot explain the translation of peripheral defocus towards protection on myopia progression, and a different explanation needs to be found.

## Introduction

Over 140 million people use contact lenses globally as regular optical wear^1^. Single vision contact lenses provide a safe and effective mean for correcting myopia, but they do fail to reduce the eye growth and the consequences that might appear with that^2,3^. The fast-growing prevalence of myopia has resulted in different optical design solutions that not only corrects the refractive error but also attempts to arrest its progression. Reported treatments are ranging from orthokeratology to bifocal or multifocal contact lenses, passing by radial refractive gradient spectacle lenses or progressive addition spectacle lenses and reported efficacies managing the progression of myopia, between 17% to 70%^4–6^. Among these novel optical treatments, multifocal contact lenses which induce defocus in the peripheral retina have shown a moderate but constant effect in slowing the eye growth^2,7^. However, whereas non-optical treatments (such as atropine) usually have their working pathways well-defined^8^; the underlying mechanisms that translates the induced peripheral defocus into a reduction of the myopia progression, are still uncharted^9^.

A closed feedback loop between the level of retinal blur and the subsequent growth of the eye is widely described in the literature (for review see Wallman and Winawer 2004)^10^. The leading hypothesis also assumes, that outside of the emmetropization process, eye growth is driven by the level of blur in the periphery^10–13^. The peripheral aberrations that comprise peripheral blur, are known to be dominated by astigmatism even when no astigmatism is present in the fovea^14,15^. This astigmatism together with comma and trefoil varies rapidly across angles while the rest of the high order aberrations remains mainly independent of the angle^16,17^. As a result of this angular dependence, asymmetrical aberrations increase with eccentricity and results in an oriented point spread function (PSF) with a decrease in the image quality. However, as a result of the reduced resolution found in the periphery^16^, the changes on the visual quality promoted by the defocus alone might be subtle and not high enough to be perceived by the visual system^18^. So, defocus alone might not explain why those contact lenses deter the progression of myopia^19^.

Two main hypotheses may still grant to a certain extent a role to defocus in the progression of myopia. The first one proposes the conflict in the spatially oriented ganglion cells^20–23^ and its selectivity that can act as myopigenic clues^24^. Thus, eye growth might be prompted in case a large anisotropy is present in the periphery^25^, and the underlying mechanism of multifocal contact lenses may be related to a reduction of the ratio of radial to azimuthal contrast^25^. Nonetheless, this anisotropic or directional blur has been reported to match neuronally, suggesting a coupling between the two systems^25–29^. Thus, this hypothesis can not explain why some contact lenses still report some effect slowing myopia even after two years of wear^7,30^. Meanwhile, another hypothesis suggests that the ebb in the image quality might be a possible indicator for the retina to react to the presence of blur, deploying signals that are related to the growth of the eye^30^. Nowadays, wave-front technologies allow us to recompose refractive errors and aberrations of the eye at different eccentricities^31^ and thus, image quality metrics can be computed readily across the visual field.

In this study, the image quality metrics while wearing contact lenses are mapped and estimated for a visual field of 20 degrees surrounding the fovea. The visual field is segmented for further analysis, and the possible implications of the image quality crumble through multifocal contact lenses are discussed in detail.

## Results

The core findings are split into three main groups: (A) the analysis of the root mean square errors (RMSE), where a single number reports the differences against the achievable limit for each image quality conditions, (B) the full-field data-sets analysis, where all the points from the maps were analysed, and finally (C) the sub-analysis by regions and groups (peripheral refractive patterns and optical states), where points are analysed by taking into account their location and the refractive pattern of the subject.

### Root mean square errors

While analysing the RMSE, the naked eye condition obtained the highest similarity to the ideal image quality (RMSE = 0.3139). The multifocal centre-near design contact lens significantly degraded the overall quality metric (RMSE = 0.3912; Mann-Whitney-U; p = 0.0010) from the naked eye, while the centre-distance design contact lens did degradate the image quality as well, but without reaching a statistical significance (RMSE = 0.3601; Mann-Whitney-U; p = 0.0183). These results were also replicated with the “Lenna image” at a 5% significance level. Table 1 provides a clear overview of the variance of the results owed to the image used, for direct comparison, the results obtained with the “Lenna image” are kept in a public repository (check Data availability section), and the choice of the “Pirate or Male image” is discussed in the Methods section.

**Table 1:** RMSE results with both images tested, under different conditions.

Image	RMSE NE	RMSE CN	RMSE CD	p (NE-CN)	p (NE- CD)
Pirate or Male image	0.3139	0.3912	0.3601	0.0010**	0.0183*
Lenna image	0.2315	0.2897	0.2720	0.0166*	0.3552

*(p < 0.05) **(p < 0.01).

### Full difference maps datasets

#### Comparison between optical conditions

**Figure 1:**
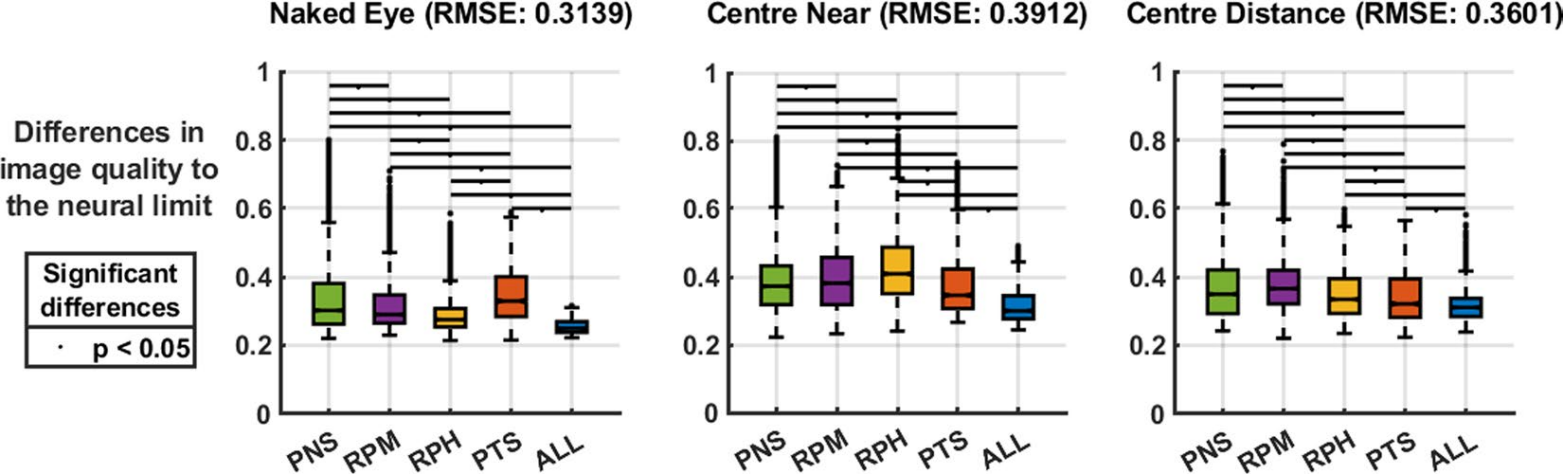
Full-data matrix comparison between peripheral refractive types, grouped by optical treatments. Overall RMSE (Root mean square errors) of each group is written in the subplot headers. Black lines on top indicate significant differences after post-hoc correction (p < 0.05). The different peripheral refractive groups analysed are positive nasal skew (PNS), relative peripheral myopia (RPM), relative peripheral hyperopia (RPH) and positive temporal skew (TPS) refraction.

The results of the full difference maps data-sets revealed that, independently of the optical condition (naked eye, centre-near or centre-distance contact lens), each classified type of peripheral refraction behaved significantly different (Kruskal-Wallis; p < 0.01) as it is shown in Fig. 1. Multiple pairwise comparisons reported also significant differences between all groups (Scheffé’s correction; p < 0.05).

#### Comparison between peripheral refractive patterns

**Figure 2:**
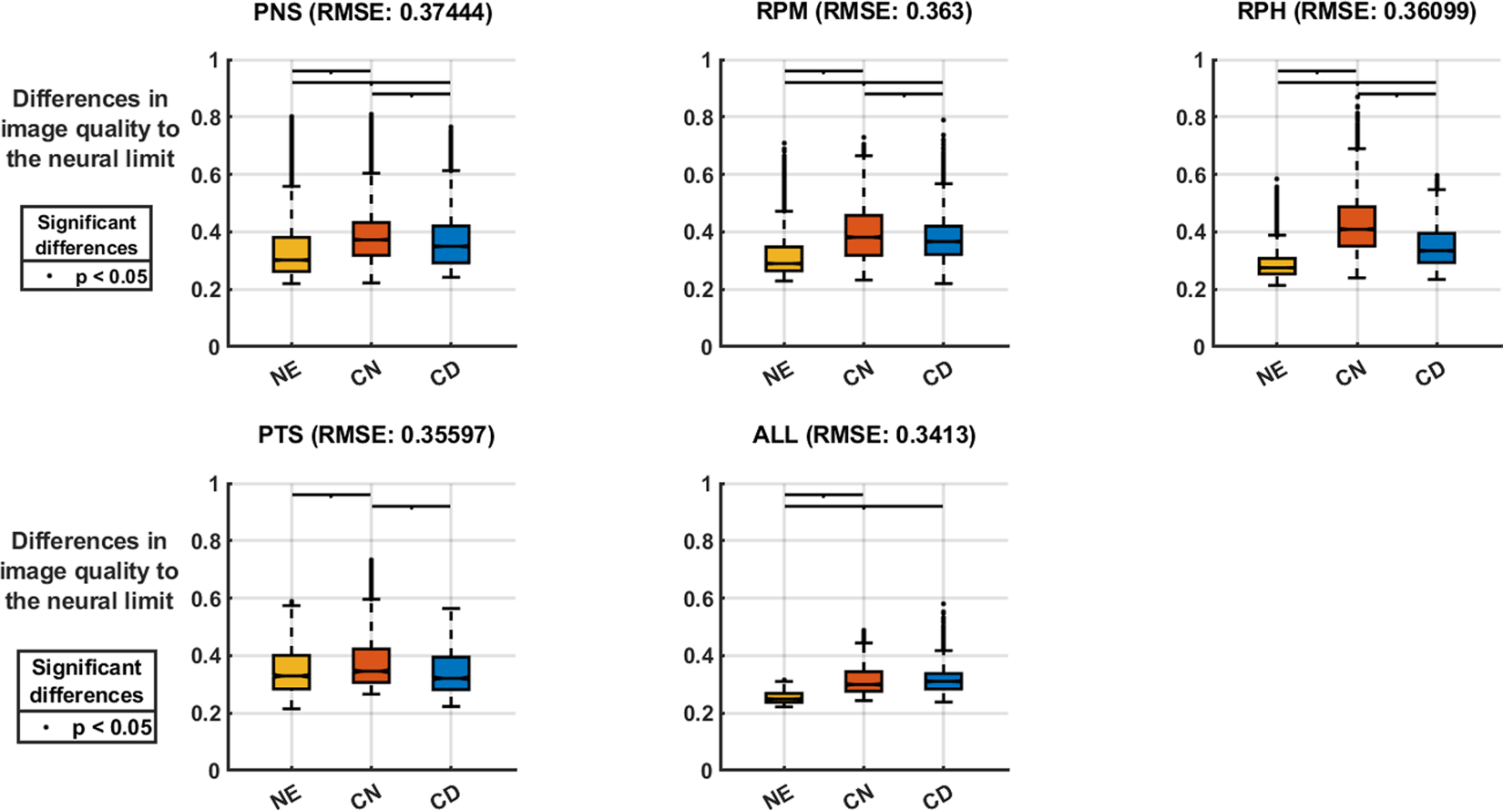
Full-data matrix comparison between optical treatments, grouped by peripheral refraction types. Overall RMSE (Root mean square errors) of each group is written in the subplot headers. Black lines on top indicate significant differences (p < 0.05).

As it can be observed in the Fig. 2, the optical treatments showed significant differences (Kruskal-Wallis; p < 0.01). However, the multiple pairwise comparisons found no difference in the image quality between the naked eye and centre-distance contact lens for the case of the positive temporal skew profile (Scheffé’s correction; p > 0.05). Additionally, no differences were found between the two contact lens conditions when the mean values of all subjects were analysed irrespectively of the group to which the subjects belonged. (Scheffé’s correction; p > 0.05).

### Regional analysis

**Figure 3:**
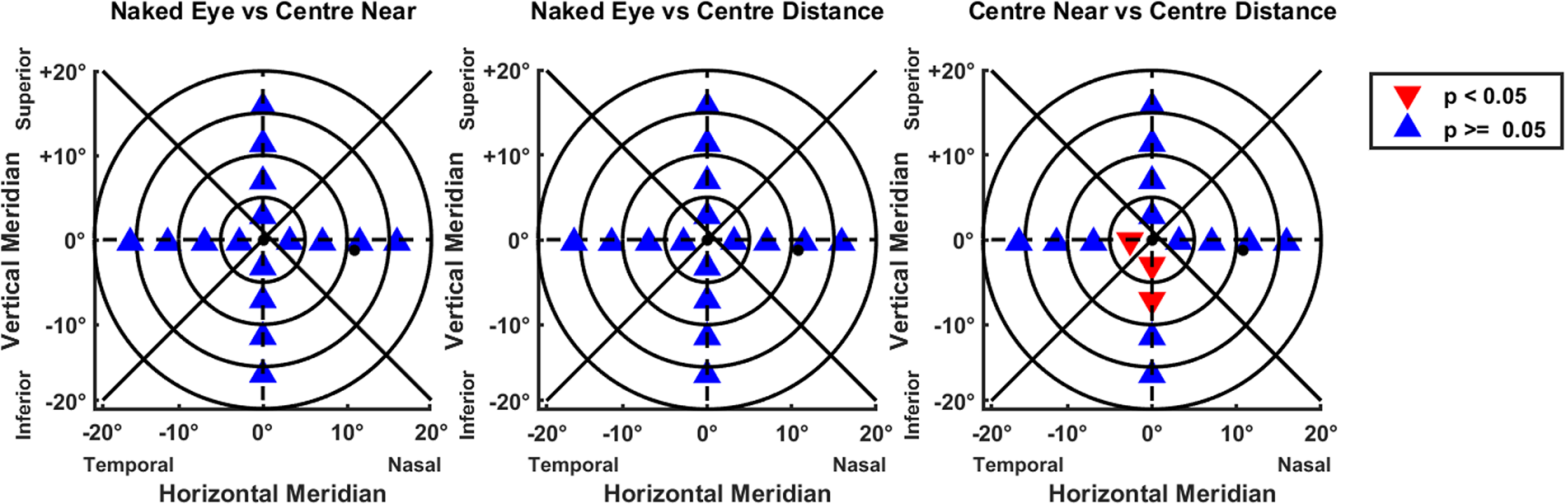
Regional segmented areas showing significant changes through multiple pairwise comparisons with Scheffé’s correction. Comparison of the different optical solutions tested. In Δ blue significant differences and in ∇ red the non-significant differences.

When segmented into regions (see Fig. 3) the quality image coefficients between the optical treatments were found to be significantly different (Kruskal-Wallis; p < 0.01), except the deterioration of the image quality in the inferior (0–10°) and temporal (0–5°) regions where it was the same, no matter which contact lens was used (Scheffé’s correction; p > 0.05).

**Figure 4:**
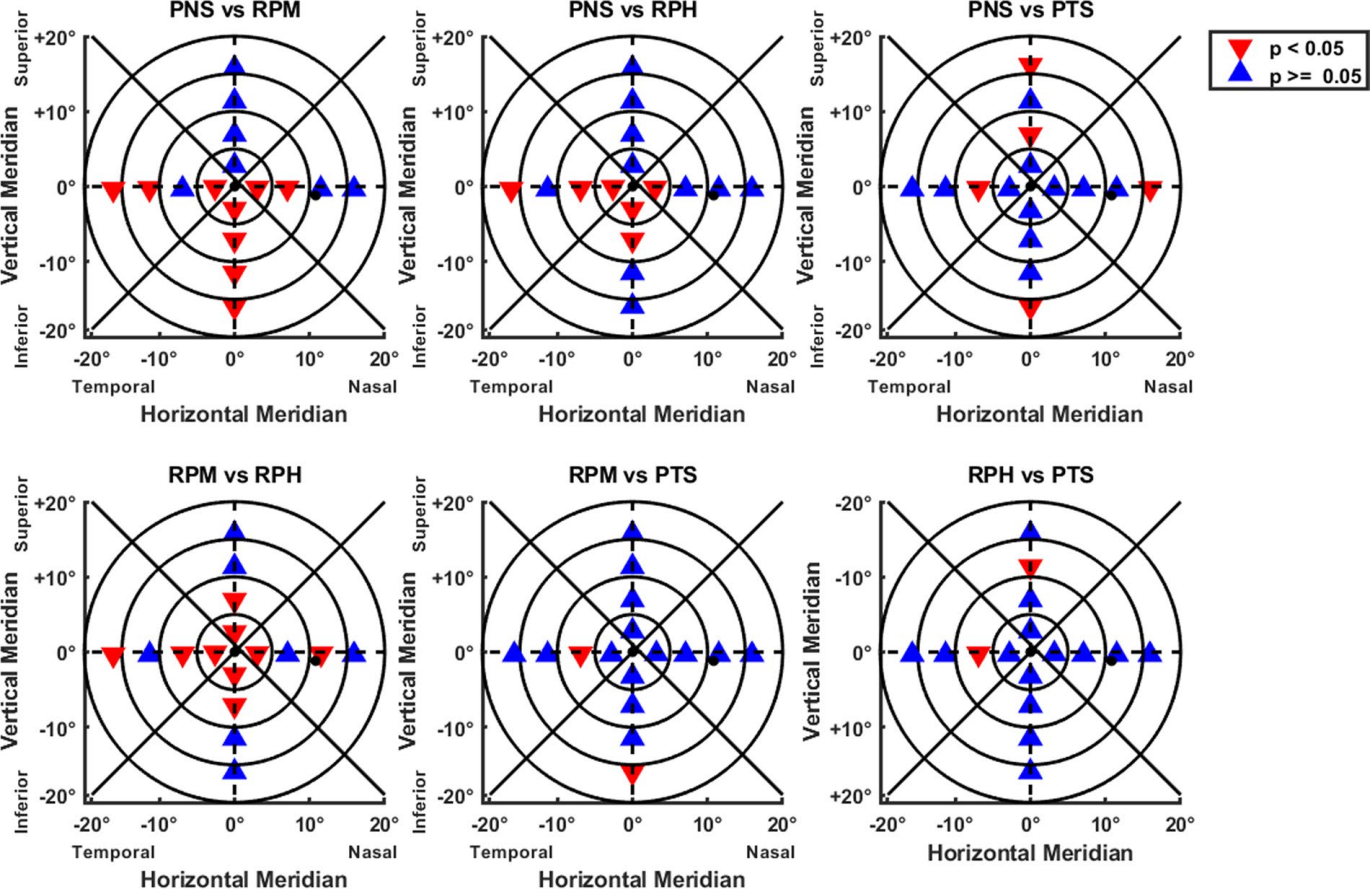
Regional segmented areas showing significant changes through multiple pairwise comparisons with Scheffé’s correction. Comparison of the different peripheral refractive groups. In Δ blue significant differences and in ∇ red the non-significant differences.

The regional comparison between the different peripheral refraction patterns presented significant differences (Kruskal-Wallis; p < 0.01), although not all the regions followed the significant differences in the pairwise regions comparisons (Scheffé’s correction; p > 0.05) as shown in the Fig. 4.

## Discussion

Currently, several optical treatments have shown a reduction in the progression of myopia, but the underlying mechanisms on which these treatments rely, still, remain unclear. As one theory supports the influence of the peripheral refractive error of the eye, the current study obtained similar results as reported earlier^32,33^. Looking beyond peripheral refraction, a global approach describing the retinal defocus patterns^34^ can explain the inter-subject variability regarding the efficacy of myopia interventions^2^. While the question remains, as to how the retina distinguishes lower order as well as higher order aberrations, a more in-depth analysis of the full image quality is required. Indicators that allow describing the image quality with such treatments will then, facilitate the understanding of the progression of myopia or the development of more efficient optical treatments to control the progression of myopia.

**Figure 5:**
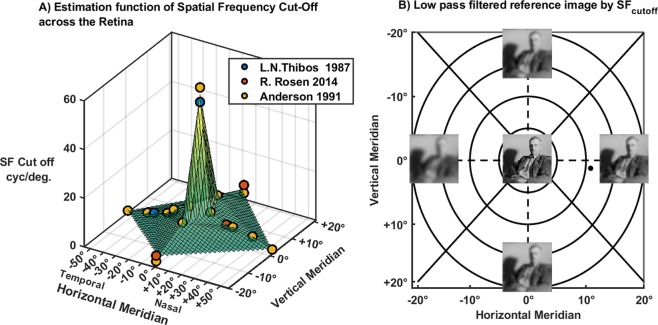
Estimation of the spatial frequency limit. (**A**) Surface plot of the function, plus the dataset from different studies included. (**B**) Representation of the reference image through a low-pass filter that restricts to the spatial frequency limit on each retinal location. For display purposes herein, only the foveal and 20° NTSI (nasal/temporal/superior/inferior) eccentricities are imaged.

**Figure 6:**
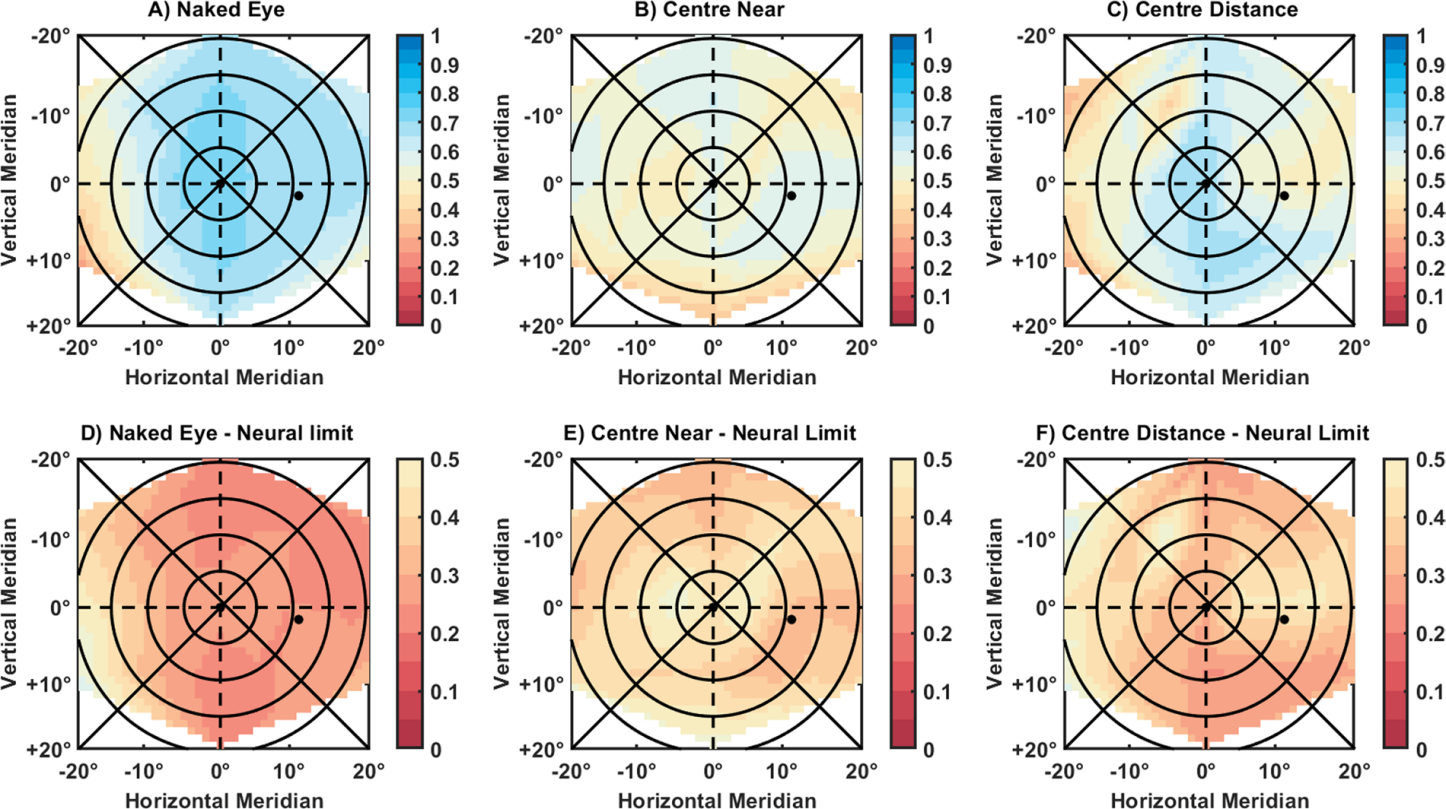
Shows the image quality across the visual field for one subject while wearing (**A**) no lens correction, (**B**) a multifocal contact lens with centre-near design and (**C**) a centre-distance contact lens design. It also shows the differences that these optical conditions caused to the image quality limit.

Image quality metrics were computed across a large visual field, not only for the naked eye but additionally for two different optical designs of multifocal contact lenses. Other authors have published prior work on image quality and contact lenses^18,35–37^, but to the best of our knowledge, this study reports for first-time results for a wider visual field (not only the horizontal meridian) and relies on real aberration measurements rather than theoretical simulations^18^.

In congruence with the published literature^38,39^, results indicate that image quality across the visual field is reduced when multifocal contact lenses are worn. However, the overall results presented some inconsistencies, depending on the analysis. On the one hand, with the RMSE full-field comparison, the image quality from the naked eye did not decay significantly when the multifocal centre-distance design was present. In contradiction, and when analysing the local or full-field grouped data, this optical design of the contact lens did promote significant differences against the naked eye.

Results of the RMSE, where only the centre-near design deteriorate the image quality, cannot explain why several authors found that centre-distance contact lenses show a better efficacy on the control of myopia progression^40,41^. Nonetheless, when assessing a more detailed test, the centre-distance design seemed also capable of promoting differences in every peripheral refractive group, except for the positive temporal skew (PTS) pattern. The absence of significant changes in this group might be the underlying reason why the RMSE comparison failed to find significant differences.

In the grouped/ranked analysis, the mean of the groups did not present differences between centre-near and centre-distance, which precludes from concluding that there are high differences in the reduction of image quality between contact lenses. The regional analysis of the different optical solutions indicated that the inferior field might be the main contributor for the non-significant changes between the different contact lens designs (as in the full-field analysis). Although no pattern can be established in the pairwise regional differences between peripheral refractive groups, it appears that image quality worsens on a similar manner for relative peripheral hyperopia (RPH), relative peripheral myopia (RPM) and positve nasal skew (PNS) groups in the central area (0 to 5 degrees). Namely, the positive nasal skew (PNS) and the relative peripheral myopia (RPM) full inferior visual field presented a similar behaviour. Contrarily to the pure defocus analysis^34^, the deterioration of the image quality when contact lenses are worn, does not seem to rely on the skewness of the peripheral refraction. Only PTS (positive temporal skew) appears to overtake the optical treatments and be less receptive to changes produced by multifocal centre-distance lenses.

The use of cross-correlation values as a full-reference image quality metric^42^ was by no means trivial. Most of the studies analysing the image quality metrics over the peripheral visual field tend to use the modulation transfer function (MTF) or the Visual Strehl ratio (VSOTF)^43,44^. While in general, these metrics are appropriate parameters to evaluate the quality of any optical system, the use of such metrics in the context of myopia research, limits the interpretability of the results, given the point that neuronal factors are not taken into account. Essentially, the retinal image quality is not only limited by the optical components but also by the distribution of the photoreceptors^45^, as well as the size and location of the receptive ganglion fields across the retina^46–48^, and both factors are known to contributing to reduce peripheral sensitivity to contrast. Interference fringes^49^ or adaptive optics systems^50^ can measure this change in the neural CSF by surpassing the optical system and testing the angular resolution on specific contrast and frequencies gratings, that are influenced by the neural factors alone. However, given the retrospective nature of this study, rather than measure the cut-off frequency of the NCSF (*f*_*cuttoff*_ NCSF) for each subject, and in every eccentricity, the values of the spatial frequency cut-off were interpolated from values reported earlier in the literature^51–53^.

The authors acknowledge several limitations to this study that needs to be mentioned. First of all, the measurements of aberrometry while subjects are wearing contact lenses can induce errors, for example due to a displacement of the contact lens. To limit the influence of the potential error input, the coefficients were averaged from four scans, and the visual field estimation was only interpolated for a smaller visual field than the measurements allows. Even though measurements were carried out while the subject fixated a target in three meters distance to avoid any accommodative effect, residual accommodative changes could happen, affecting the image quality as well^54^. The number of subjects may have limited the definition of the peripheral power profiles (especially in the case of PTS) and the conclusions of the grouped analysis, since some groups were represented only by few subjects. Furthermore, in this study, only two contact lenses were tested. Due to the differences in the optical designs of multifocal contact lenses from different manufacturers, this limitation may prevent the findings to be extrapolated to contact lenses from other suppliers. Measurements of the neural contrast sensitivity function (NCSF) in the periphery that are reported in the literature are limited just to a few subjects or a few eccentricities^36,52^. This limitation occurs since such measurements are exhaustive and time consuming for the subject. Given the fact that spatial frequency cut-offs were extrapolated from the literature rather than real measurements for each subject, the obtained results might be less precise. However, the piecewise linear surface function that defines the spatial frequency cut-off can be improved in future studies, by adding more values.

To conclude, regional and groups segmentation of the data can prevent the misinterpretation of results. Hence, the analysis of root mean square errors (RMSE) or the simplification of the image quality over the whole retinal field to a single number can lead to controversial results, and should therefore not be recommended as a benchmark. Although slight differences are shown between contact lenses, the differences are small and not constant over the different analysed segments or the different conducted analysis. On the other hand, image quality decrease caused by multifocal contact lenses alone does not seem to be the underlying reason for the myopia control that these contact lenses can achieve. Future research needs to study other hypotheses to explain how multifocal contact lenses achieve myopia control.

## Methods

### Data acquintance

Earlier acquired data from thirteen subjects were retrospectively analysed^34^. On average, the subjects had a spherical equivalent error (SE) of −3.25 Dioptres (−0.75 to −6.50 Dioptres) and an axial length of 25.37 mm (22.55 to 26.50 mm). The ethics authorisation to perform the measurements was granted by the Research Ethics Committee from the University of Murcia. ID: 1108/2015. The study adhered to the principles of the Helsinki declaration (1998) and posterior amends and written informed consent was obtained from the subjects prior to the investigation. All data was store and analyse in in full compliance to the principles of the Data Protection Act GDPR 2016/679 of the European Union^55^.

Refraction from the right eyes (OD) of the subjects were objectively measured using a Hartmann-Shack sensor (VAO device, AOVIS-1, VOptica SL, Murcia, Spain)^56,57^ and subjectively measured using the same device by a trained optometrist (author MGG). Axial length was obtained using a standard optical biometer (Lenstar LS900; Haag-Streit AG, Köniz, Switzerland). All the subjects were usual contact lens wearers with spherical equivalent refractive errors below −7 dioptres and without any medical or ocular pathologies record.

Measurements were performed with no lens in front of the eye (naked eye) and while wearing two different optical designs of multifocal contact lenses, one with centre-near design and one with the centre-distance design. The contact lens used in this study were the hydrogel Xtensa, Filcon IV 1 55%, by Mark’ennovy, with centre-near and centre-distance optical designs. The central refractive power was −0.25 dioptres for the distance focus and an additional power of +2.25 and +2.50 dioptres for the near-centre and distance-centre designs respectively. Further information regarding these contact lenses can be found here^34^.

The aberration coefficients of the right eyes were recorded using an open-view peripheral wave-front sensor (Voptica SL, Murcia, Spain) as described by Jaeken *et al*.^35,58^. During the measurements, subjects were fixating a laser target in 3 meters distance while the instrument scanned over a wide range of horizontal arc (80°); recording 81 high-resolution Hartmann-Shack (HS) images within 1.8 seconds. The obtained images were processed up to the sixth order of Zernike polynomials with a 3 mm pupil diameter, rendering full aberrometry data for every point by using the software provided by the manufacturer.

After measuring the mean horizontal meridian or equator, the subjects were asked to fixate at +10°, +20°, −10° and −20 degrees. Four scans (equivalent to 324 HS images) in each vertical fixation point were averaged to define the peripheral refraction and aberrometry profiles.

### Data analysis

The point spread function was computed using Matlab r2018a (The MathWorks Inc, Natick, MA, USA) for every point (in 5-degrees stepwise), using the Zernike coefficients obtained within the wavefront measurements. In all the computations, low order aberrations (defocus, oblique and vertical astigmatism) were normalised to the error found in the fovea^16^, being the remaining coefficients computed at the wavelength of 532 nm.

Similarly to the theoretical approach from Ji *et al*.^18^, a grey-scaled 512 × 512 pixels image was used as a reference image. The image was located subtending 2 degrees on every retinal location, and the resolution of the same was 0.2344 m/pixel. At each peripheral angle, this reference image was convolved with the point spread function (PSF). After this, convolved images passed through a low-pass filter to match the image with the spatial frequency domain where the eye can still operate for the corresponding eccentricity. See Figs. 5 and 6.

Owing to the controversial origin of ‘Lenna or Lena’s image’^59^, in this study, the so known as ‘Pirate or Male’ image (5.3.01 from the SIPI image database^60^) was used. This image is according to the literature^61^ the most similar picture in terms of features. Nevertheless, owing to the natural variation of the outcomes that using a different image causes, the results obtained with the ‘Lena’ model remain in the repository to allow a direct comparison with the previous study from Ji *et al*.^18^.

Given the retrospective nature of this study, rather than measuring the cut-off frequency of the NCSF (*f*_*cuttoff*_ *NCSF*) for each subject, and in every eccentricity, the values of the spatial frequency cut-off were interpolated from values that were reported in the literature before^51–53^. Due to the lack of values for all the required locations, a piecewise linear surface function^62,63^ was fitted using the 20 coefficients reported in the literature. Then, using the vertical and horizontal position, an estimation of the cut-off limit was gained and used to match the resolution limit of the convolved images through a 2D Gaussian low-pass filter^64,65^.

Following, the filtered images were cropped to avoid any aliasing effect on the edges^66^ and subsequently compared with the original image. Two-dimensional cross-correlation metrics were assessed objectively by allocating Pearson correlation coefficients for every comparison^67^. The resulting coefficients ranged from 0 to 1, where 1 refers to the highest similarity between the images and therefore the best image quality, and 0 to the worse image quality.

On an individual basis (for each subject and each condition), the obtained 2D-cross correlation values were used to interpolate the residuals of the surface fit, covering a range of approximately 40° × 40° (resulting in a matrix/map of 40 × 40 pixels with cross-correlation values at every degree).

Given the neural limitation in the periphery of the eye and the diffraction limit, certain eccentricities would never reach the perfect image quality metric. A direct comparison of the convolved and filtered image against the reference image could provide meaningful information regarding the image quality deterioration due to the peripheral neural factors. Those limitations imposed by the neural factors are not equal in all the retinal locations. Therefore, a more insightful value of how the optical treatment changes the whole field can be observed comparing the difference between the neural limit and the optical paths rather than a direct comparison of the image quality ebb. For this reason, final matrices were remapped with the same spatial distribution and resolution but revealing the changes caused by optical conditions in relation to the neural boundary. These values were achieved by subtracting the two-dimensional cross-correlation values from the neural limited cross-correlation matrix.

### Segmentation and grouping

To achieve a more detailed analysis regarding the image quality and to take its location across the visual field in consideration, the significance maps were segmented following a 45° nasal/temporal/superior/inferior (NTSI) angular division with a complementary annular division every five degrees. Smaller sections resulted in sets of sixteen points while the most larger sections contained above one hundred and forty values.

Moreover, the final maps from every subject were classified attending to their peripheral refractive pattern, under naked eye conditions. Four groups were established, based on the nasal and temporal outer edges compared to the mean refraction in the central area. The profiles were classified as nasal positively skewed (PNS; n = 5), relative peripheral myopia (RPM; n = 4), relative peripheral hyperopia (RPH; n = 3) and temporal positive skewed (PTS; n = 1).

### Flow diagram

**Figure 7:**
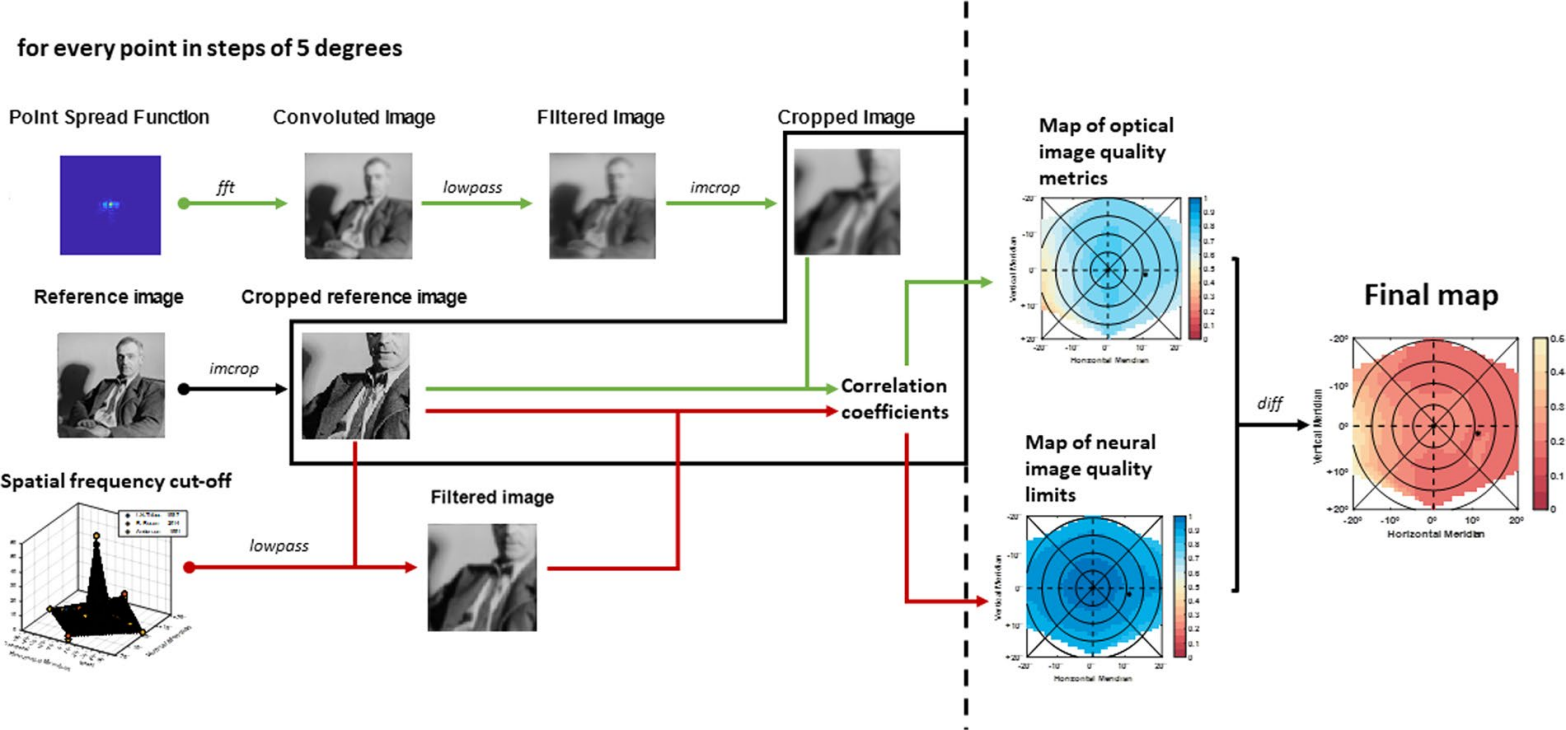
Flow chart demonstrating the process followed to calculate the coefficient maps and the final maps.

The scheme in Fig. 7 illustrates how the significance maps were built from the aberrations that were measured with the open-view peripheral Hartmann-Shack sensor and the theoretical resolution limit from the neural factors.

### Statistical analysis

The differences between the 2-D significance maps and the limiting spatial frequency cut-off map were used to quantify the overall differences produced by each condition for every subject. The root mean square errors (RMSE) as a single representative value of the full visual field as well as the full-populated maps were analysed independently. The non-parametric Mann-Withney-U test was used to test the RMSE from the contact lens conditions against the naked eye. Non-parametric Kruskal-Wallis with multiple pairwise comparisons were performed for the final maps, to test the null hypothesis that there were no differences between groups.

For the analysis of the segmented areas, non-parametric Kruskal-Wallis tests and multiple pairwise comparisons with the Scheffé’s correction were performed. Unlike Bonferroni correction, this procedure provides a simultaneous confidence level for comparisons of all linear combinations of the means, and it is conservative for comparisons of simple differences of pairs^68^.

## Data Availability

Public repository (https://gin.g-node.org/Miguel/SREP-19-15078) on GIN holds all the necessary data to replicate the results. Github repository (https://github.com/mikelgg93/FIG_EstimationSF.git) contains the function used to obtain the spatial frequency limit.

## References

[1] Cope JR (2015). Contact LensWearer Demographics and Risk Behaviors for Contact Lens-Related Eye Infections. MMWR Morb Mortal Wkly Rep.

[2] Huang J (2016). Efficacy Comparison of 16 Interventions for Myopia Control in Children: A Network Meta-analysis. Ophthalmology.

[3] Flitcroft DI (2012). The complex interactions of retinal, optical and environmental factors in myopia aetiology. Prog. retinal eye research.

[4] Wolffsohn JS (2016). Global trends in myopia management attitudes and strategies in clinical practice. Contact Lens Anterior Eye.

[5] Smith MJ, Walline JJ (2015). Controlling myopia progression in children and adolescents. Adolesc. health, medicine therapeutics.

[6] Walline, J. J. *et al*. Interventions to slow progression of myopia in children. *Cochrane Database Syst. Rev*., 10.1002/14651858.CD004916.pub3 (2011). 10.1002/14651858.CD004916.pub3 PMC4270373 22161388

[7] Walline JJ, Greiner KL, McVey ME, Jones-Jordan LA (2013). Multifocal Contact Lens Myopia Control. Optom. Vis. Sci..

[8] Chakraborty, R. & Pardue, M. T. *Molecular and Biochemical Aspects of the Retina on Refraction*., vol. 134, 1 edn (Elsevier Inc., 2015). 10.1016/bs.pmbts.2015.06.013 PMC5588150 26310159

[9] Smith EL, Campbell MCW, Irving E (2013). Does peripheral retinal input explain the promising myopia control effects of corneal reshaping therapy (CRT or ortho-K) & multifocal soft contact lenses?. Ophthalmic Physiol. Opt..

[10] Wallman J, Winawer J (2004). Homeostasis of eye growth and the question of myopia. Neuron.

[11] Read SA, Collins MJ, Sander BP (2010). Human Optical Axial Length and Defocus. Investig. Opthalmology & Vis. Sci..

[12] Schaeffel F, Wildsoet C (2013). Can the retina alone detect the sign of defocus?. Ophthalmic Physiol. Opt..

[13] Neil Charman W, Radhakrishnan H (2010). Peripheral refraction and the development of refractive error: a review. Ophthalmic Physiol. Opt..

[14] Gustafsson J, Terenius E, Buchheister J, Unsbo P (2001). Peripheral astigmatism in emmetropic eyes. Ophthalmic & physiological optics: journal Br. Coll. Ophthalmic Opt. (Optometrists).

[15] Lundström L (2007). Effect of optical correction and remaining aberrations on peripheral resolution acuity in the human eye. Opt. Express.

[16] Jaeken B, Artal P (2012). Optical Quality of Emmetropic and Myopic Eyes in the Periphery Measured with High-Angular Resolution. Investig. Opthalmology & Vis. Sci..

[17] Navarro R, Moreno E, Dorronsoro C (1998). Monochromatic aberrations and point-spread functions of the human eye across the visual field. J. Opt. Soc. Am. A, Opt. image science, vision.

[18] Ji Q, Yoo YS, Alam H, Yoon G (2018). Through-focus optical characteristics of monofocal and bifocal soft contact lenses across the peripheral visual field. Ophthalmic Physiol. Opt..

[19] Atchison DA, Rose R (2016). The Possible Role of Peripheral Refraction in Development of Myopia..

[20] Blakemore C, Campbell FW (1969). On the existence of neurones in the human visual system selectively sensitive to the orientation and size of retinal images. The J. physiology.

[21] Passaglia CL, Troy JB, Rüttiger L, Lee BB (2002). Orientation sensitivity of ganglion cells in primate retina. Vis. Res..

[22] Antinucci, P. & Hindges, R. Orientation-Selective Retinal Circuits in Vertebrates. *Front. Neural Circuits* **12**, 10.3389/fncir.2018.00011 (2018). 10.3389/fncir.2018.00011 PMC5808299 29467629

[23] Antinucci P, Abbas F, Hunter PR (2016). Orientation Selectivity in the Retina: ON Cell Types and Mechanisms. J. Neurosci..

[24] Charman WN (2011). Keeping the world in focus: how might this be achieved?. Optom. vision science: official publication Am. Acad. Optom..

[25] Zheleznyak L, Barbot A, Ghosh A, Yoon G (2016). Optical and neural anisotropy in peripheral vision. J. Vis..

[26] Frisén L, Glansholm A (1975). Optical and neural resolution in peripheral vision. Investig. ophthalmology.

[27] Ghosh A, Zheleznyak L, Barbot A, Jung H, Yoon G (2017). Neural adaptation to peripheral blur in myopes and emmetropes. Vis. Res..

[28] Artal P (2004). Neural compensation for the eye’s optical aberrations. J. Vis..

[29] Venkataraman AP (2019). Peripheral resolution and contrast sensitivity: effects of monochromatic and chromatic aberrations. J. Opt. Soc. Am. A.

[30] Sun Y (2015). Orthokeratology to control myopia progression: A meta-analysis. PLOS ONE.

[31] Fedtke C, Ehrmann K, Holden BA (2009). A Review of Peripheral Refraction Techniques. Optom. Vis. Sci..

[32] Seidemann A, Schaeffel F, Guirao A, Lopez-Gil N, Artal P (2002). Peripheral refractive errors in myopic, emmetropic, and hyperopic young subjects. J. Opt. Soc. Am. A.

[33] Lundström L (2009). Peripheral optical errors and their change with accommodation differ between emmetropic and myopic eyes. J. vision.

[34] García García Miguel, Pusti Dibyendu, Wahl Siegfried, Ohlendorf Arne (2019). A global approach to describe retinal defocus patterns. PLOS ONE.

[35] Rosén R (2012). Evaluating the peripheral optical effect of multifocal contact lenses. Ophthalmic Physiol. Opt..

[36] Shen Jie (2014). Ocular Aberrations and Image Quality, Contact Lens and MYOPIA Progression. Ophthalmology - Current Clinical and Research Updates.

[37] Charman W, Walsh G (1986). Retinal image quality with different designs of bifocal contact lens. J. The Br. Contact Lens Assoc..

[38] Kollbaum PS, Jansen ME, Tan J, Meyer DM, Rickert ME (2013). Vision Performance With a Contact Lens Designed to Slow Myopia Progression. Optom. Vis. Sci..

[39] Fedtke C, Ehrmann K, Thomas V, Bakaraju RC (2016). Visual performance with multifocal soft contact lenses in non-presbyopic myopic eyes during an adaptation period. Clin. Optom..

[40] Berntsen DA, Kramer CE (2013). Peripheral defocus with spherical and multifocal soft contact lenses. Optom. vision science: official publication Am. Acad. Optom..

[41] Aller TA, Liu M, Wildsoet CF (2016). Myopia Control with Bifocal Contact Lenses. Optom. Vis. Sci..

[42] Zhang, L., Zhang, L., Mou, X. & Zhang, D. *A comprehensive evaluation of full reference image quality assessment algorithms*. 11–14 (2012).

[43] Applegate RA, Marsack JD, Thibos LN (2006). Metrics of retinal image quality predict visual performance in eyes with 20/17 or better visual acuity. Optom. vision science: official publication Am. Acad. Optom..

[44] Shen J, Thibos LN (2011). Peripheral aberrations and image quality for contact lens correction. Optom. vision science:official publication Am. Acad. Optom..

[45] Curcio CA, Sloan KR, Kalina RE, Hendrickson AE (1990). Human photoreceptor topography. The J. Comp. Neurol..

[46] Watson AB (2014). A formula for human retinal ganglion cell receptive field density as a function of visual field location. J. Vis..

[47] Croner LJ, Kaplan E (1995). Receptive fields of P and M ganglion cells across the primate retina. Vis. Res..

[48] Cho MW, Choi M (2014). A model for the receptive field of retinal ganglion cells. Neural Networks.

[49] Campbell FW, Green DG (1965). Optical and retinal factors affecting visual resolution. The J. physiology.

[50] Marcos S (2017). Vision science and adaptive optics, the state of the field. Vis. Res..

[51] Thibos LN, Walsh DJ, Cheney FE (1987). Vision beyond the resolution limit: Aliasing in the periphery. Vis. Res..

[52] Rosen R, Lundstrom L, Venkataraman AP, Winter S, Unsbo P (2014). Quick contrast sensitivity measurements in the periphery. J. Vis..

[53] Anderson SJ, Mullen KT, Hess RF (1991). Human peripheral spatial resolution for achromatic and chromatic stimuli: limits imposed by optical and retinal factors. The J. Physiol..

[54] López-Gil N (2008). Accommodation-Related Changes in Monochromatic Aberrations of the Human Eye as a Function of Age. Investig. Opthalmology & Vis. Sci..

[55] The European Parliament and the Council of the European Union. Regulation (EU) 2016/679 of the European Parliament and of the Council. *Off. J. Eur. Union* 59 (2016).

[56] Fernández EJ, Manzanera S, Piers P, Artal P (2002). Adaptive optics visual simulator. J. refractive surgery (Thorofare, N.J.: 1995).

[57] Hervella L, Villegas EA, Prieto PM, Artal P (2019). Assessment of subjective refraction with a clinical adaptive optics visual simulator. J. Cataract. & Refract. Surg..

[58] Jaeken B, Lundström L, Artal P (2011). Fast scanning peripheral wave-front sensor for the human eye. Opt. Express.

[59] Board E (2018). A note on the Lena image. Nat. Nanotechnol..

[60] The USC-SIPI Image Database, http://sipi.usc.edu/database/database.php.

[61] Board E (2017). On alternatives to Lenna. J. Mod. Opt..

[62] Vieth E (1989). Fitting piecewise linear regression functions to biological responses. J. applied physiology (Bethesda, Md.: 1985).

[63] Jimenez-Fernandez VM (2016). Transforming the canonical piecewise-linear model into a smooth-piecewise representation. SpringerPlus.

[64] Gonzalez, R. C., Woods, R. E. R. E. & Eddins, S. L. *Digital Image processing using MATLAB* (Pearson Prentice Hall, 2004).

[65] Dogra A, Bhalla P (2014). Image Sharpening By Gaussian And Butterworth High Pass Filter. Biomed. Pharmacol. J..

[66] Thibos LN, Still DL, Bradley A (1996). Characterization of spatial aliasing and contrast sensitivity in peripheral vision. Vis. Res..

[67] Kim MJ, Zheleznyak L, MacRae S, Tchah H, Yoon G (2011). Objective evaluation of through-focus optical performance of presbyopia-correcting intraocular lenses using an optical bench system. J. Cataract. Refract. Surg..

[68] Scheffé, H. *The Analysis of Variance* (Wiley-Interscience Publication, 1959).

